# Medical costs, Cesarean delivery rates, and length of stay in specialty hospitals vs. non-specialty hospitals in South Korea

**DOI:** 10.1371/journal.pone.0188612

**Published:** 2017-11-30

**Authors:** Seung Ju Kim, Sun Jung Kim, Kyu-Tae Han, Eun-Cheol Park

**Affiliations:** 1 Department of Nursing, College of Nursing, Eulji University, Seongnam, Republic of Korea; 2 Department of Health Administration, Soonchunhyang University, Chungnam, Republic of Korea; 3 Department of Policy Research Affairs, National Health Insurance Service Ilsan Hospital, Ilsan, Republic of Korea; 4 Institute of Health Services Research, Yonsei University, Seoul, Republic of Korea; 5 Department of Preventive Medicine, Yonsei University College of Medicine, Seoul, Republic of Korea; Yokohama City University, JAPAN

## Abstract

**Background:**

Since 2011, specialty hospitals in South Korea have been known for providing high- quality care in specific clinical areas. Much research related to specialty hospitals and their performance in many such areas has been performed, but investigations about their performance in obstetrics and gynecology are lacking. Thus, we aimed to compare specialty *vs*. non-specialty hospitals with respect to mode of obstetric delivery, especially the costs and length of stay related to Cesarean section (CS) procedures, and to provide evidence to policy-makers for evaluating the success of hospitals that specialize in obstetric and gynecological (OBGYN) care.

**Methods:**

We obtained National Health Insurance claim data from 2012 to 2014, which included information from 418,141 OBGYN cases at 214 hospitals. We used a generalized estimating equation model to identify a potential association between the likelihood of CS at specialty hospitals compared with other hospitals. We also evaluated medical costs and length of stay in specialty hospitals according to type of delivery.

**Results:**

We found that 150,256 (35.9%) total deliveries were performed by CS. The odds ratio of CS was significantly lower in specialty hospitals (OR: 0.95, 95% CI: 0.93–0.96compared to other hospitals Medical costs (0.74%) and length of stay (1%) in CS cases increased in specialty hospitals, although length of stay following vaginal delivery was lower (0.57%) in specialty hospitals compared with other hospitals.

**Conclusions:**

We determined that specialty hospitals are significantly associated with a lower likelihood of CS delivery and shorter length of stay after vaginal delivery. Although they are also associated with higher costs for delivery, the increased cost could be due to the high level of intensive care provided, which leads to improve quality of care. Policy-makers should consider incentive programs to maintain performance of specialty hospitals and promote efficiency that could reduce medical costs accrued by patients.

## Introduction

Over the past decade, the number of health care facilities in Korea has increased from 61,776 in 2000 to 84,971 in 2013 [1]. This dramatic change in the number of hospitals has coincided with political changes; as a result, the level of competition among these facilities has increased [2,3]. Increased competition has led to more profit-seeking by many hospitals and lower quality of care for patients [2,4,5]. An alternative strategy for surviving the competitive environment emerged in the form of specialty hospitals.

In Korea, the Ministry of Health and Welfare created the designation “specialty hospital” in November 2011 to promote the success of small hospitals through their specialization in certain clinical fields. Eighteen specialty areas were selected, including obstetrics and gynecology (OB/GYN), neurosurgery, and cardiovascular health, among others [3]. To be designated as a specialty hospital, the hospital must meet the Ministry of Health and Welfare selection criteria, such as a certain number of beds, physicians, and medical service departments. In addition, inpatient volume must be greater than the 30th percentile among total hospitals, and the ratio of specialty area inpatients to total inpatients must be above a certain percentage. The evaluation for designation as a specialty hospital is performed every 3 years; however, there is currently no incentive for specialty hospitals to improve or maintain their performance level at the time of evaluation.

Previous research suggests that specialty hospitals provide high-quality care at a low cost due to their skillful physicians and specialized health services [6–9]. Because specialty hospitals focus on specific areas and provide more options for specialized care, they are often perceived as providing greater quality of care compared to other hospitals [10,11]. However, it has also been suggested that they actually provide lower quality of care at a higher cost. [12–14]. In Korea, a few studies have shown that specialty hospitals are associated with high-quality care [3,15,16], although these studies were performed using small population sizes and only focused on a few clinical areas. Less is known about the performance of specialty hospitals with respect to OBGYN care, especially the rate of Cesarean section (CS) deliveries, length of patient stay, and incurred medical costs.

The CS delivery rate in Korea was 36.0% in 2013, which is the highest rate among countries in the Organization for Economic Cooperation and Development (average of 27.6% per 100 live births) [17], and has not changed significantly in the past few years. One possible cause of the high CS rate could be associated with different reimbursement rates for different modes of delivery. The average cost of a CS delivery is at least two times higher than that for a vaginal delivery and may require the health care provider to perform additional, and perhaps unnecessary, procedures if the physician lacks specialized OBGYN knowledge [18]. However, it is unknown if similar results would be observed with respect to hospitals that specialize in OBGYN care.

Thus, the aim of our study was to compare specialty *vs*. non-specialty hospitals with respect to CS rate, length of post-delivery stay, and medical costs associated with OBGYN care, in order to provide evidence for evaluating the performance of specialty hospitals to policy-makers.

## Materials and methods

### Data collection

To investigate the performance of specialty hospitals, we obtained the National Health Insurance (NHI) claim data from 2012 to 2014, which included information from 418,141 cases at 214 hospitals. We excluded unsuitable cases from diagnosis-related groups (DRG) based on payment type because the data could be impacted by the reimbursement system. The reimbursement for CS was applied to the DRG-based payment system in July 2012, which became mandatory in general hospitals and clinics on July 1, 2013. As a result, we excluded the general hospital cases from July 2012 to June 2013 in order to remove the effects of the DRG system. We also adjusted for the year to reduce different period effects of the hospitals in our study. In addition, we excluded cases from clinics and tertiary hospitals (due to their smaller sample sizes) and patients with medical aid because they are not part of the DRG system in Korea.

Ultimately, we included patient admission data from July 2012 to June 2014. We selected CS delivery (DRG codes O0160 and O0170) and vaginal delivery (DRG codes O0200 and O0299 for primiparous and multiparous patients, respectively) cases from the data. Each code was subdivided by the severity of complication(s) and comorbidity(ies) (patient clinical complexity level: PCCL/ 0 = no CCL, 1 = minor CCL, 2 = moderate CCL, 3 = severe CCL). A total of 418,141 hospitalizations at 214 hospitals were included in our analysis (CS delivery: 150,256; vaginal delivery: 267,885).

### Variables

Hospitals were sorted into one of three groups (hospital, specialty hospital, general hospital). Thirteen hospitals were designated as specialty hospitals in OBGYN. The outcome variables included method of delivery (vaginal = 0 or CS = 1), medical cost, and length of stay (LOS). We did not consider additional factors, such as previous CS or induced labor, fetal stress, or prolapse, due to the limitations related to our data. We used a binary variable to evaluate the likelihood of CS delivery according to hospital type. Medical cost was evaluated by calculating total cost (patient and insurer cost) according to delivery type, but did not consider non-payment cases because they were not included in our data. We adjusted for different costs between hospitals and general hospitals, as reimbursement rates were higher in general hospitals [2]. The LOS was measured using the patients’ date of admission and date of discharge. We used a log transformation for LOS to reflect the original scale of skewed data and to measure changes in the dependent variable in response to percentage changes in the explanatory variable [19–22].

Hospital characteristics, such hospital location (e.g., urban, rural), number of beds, and human resources (e.g., numbers of doctors and nurses, proportion of specialists) were included in the analysis. To minimize the confounding effects of differences across hospitals, we adjusted for the proportion of OBGYN patients per hospital. Patient characteristics included in the analysis were patient ID, parity (primiparous, multiparous), age, and PCCL. The LOS was adjusted only with respect to medical cost because of their direct association.

### Ethical consideration

This study used encrypted data which were not available to identify personal information. Therefore, ethics approval and informed consent were not required for this study.

### Statistical analysis

The distribution of each categorical variable was examined by an analysis of frequencies and percentages, and χ^2^ tests were performed to examine associations with CS delivery. Analysis of variance was also performed to compare the average values and standard deviations for continuous variables. A generalized estimating equation (GEE) model was used to evaluate the effects of specialty hospitals on the likelihood of CS and patient LOS. In the GEE models, the correlation of the measurements is accounted through a robust covariance matrix [23]. This model assumed proper distributions for each hospitalization case while taking into account the correlation among cases within the hospitals. Therefore, it is possible to estimate a more efficient estimator of regression parameters, and it is a major benefit to produce a reasonably accurate standard error [24]. The correlation structure was modeled as exchangeable correlation to determine the repeated outcome measurement of delivery [24,25]. GEE models provide a quasi-likelihood under the independence model criterion (QIC), which was used to assess the model’s goodness-of-fit. A lower QIC value indicated a better-fit model. We used a gamma generalized linear model based on the log link function to evaluate medical cost differences according to hospital type. In addition, subgroup analyses were performed based on patient age (≤ or > 35 years old) because age is an influencing factor in CS deliveries [26]. All statistical analyses were performed using SAS version 9.3 (SAS Institute, Inc.; Cary, NC, USA). P-values of < 0.05 indicated statistical significance.

## Results

The data used in this study consisted of 418,141 cases at 214 hospitals (n = 13 specialty hospitals; n = 136 hospitals; n = 65 general hospitals). CS deliveries (n = 150,256) accounted for 35.9% of total deliveries. The frequency of CS cases was lowest in specialty hospitals (34.4%), while the highest frequency (46.7%) was observed in general hospitals (Table 1).

**Table 1:** General characteristics of participants and hospital.

(Unit: N/M, %/SD)							
	Delivery				Total		P-value
	Cesarean delivery		Vaginal delivery				
***Main interest(n = 214)***							
**Type of hospital**							
Specialty hospital (n = 13)	28,270	(34.4)	53,997	(65.6)	82,267	(19.7)	<.0001
Hospital (n = 136)	107,160	(35.2)	196,957	(64.8)	304,117	(72.7)	
General hospital (n = 65)	14,826	(46.7)	16,931	(53.3)	31,757	(7.6)	
***Outcome variables***							
**Medical Cost**^1^	1,636,923	±221,727	1,033,811	±191,089	1,274,588	± 382,154	<.0001
**LOS**	6.54	± 1.16	3.32	± 0.94	4.49	± 1.86	<.0001
***Hospital characteristics***							
**Hospital location**							
Urban (n = 208)	149,246	(35.9)	266,836	(64.1)	416,082	(99.5)	<.0001
Rural(n = 7)	1,010	(49.1)	1,049	(51.0)	2,059	(0.5)	
**Number of Beds**	274.36	± 293.13	261.23	± 284.67	266.99	± 289.25	0.6424
**Number of doctors**	58.55	± 88.32	55.44	± 88.70	56.49	± 87.00	0.7197
**Proportion of specialist**	45.92	± 33.88	47.37	± 33.66	46.73	± 33.85	0.6722
**Number of nurses**	117.58	± 172.96	110.42	± 172.89	112.87	± 170.79	0.6599
**Proportion of patients per hospital**	66.98	± 32.68	67.53	± 31.78	66.74	± 32.27	0.8627
***Patient characteristics***							
**Parity**							
Primipara	67,093	(32.3)	140,717	(67.7)	207,810	(49.7)	<.0001
Multipara	83,163	(39.5)	127,168	(60.5)	210,331	(50.3)	
**Age**	32.11	± 4.01	30.96	± 3.84	31.44	± 3.97	<.0001
**PCCL**							
0	95,303	(30.0)	222,056	(70.0)	317,359	(75.9)	<.0001
1	33,397	(48.0)	36,196	(52.0)	69,593	(16.6)	
2	20,263	(68.6)	9,268	(31.4)	29,531	(7.1)	
3	1,293	(78.0)	365	(22.0)	1,658	(0.4)	
**Year**							
2012.07~2013.06	67,371	(34.7)	126,566	(65.3)	193,937	(46.4)	<.0001
2013.07~2014.07	82,885	(37.0)	141,319	(63.0)	224,204	(53.6)	
**Total**	150,256	(35.9)	267,885	(64.1)	418,141	(100.0)	

PCCL: patient clinical complexity level

LOS: length of stay

^**1**^Unit: KRW

**Fig 1:**
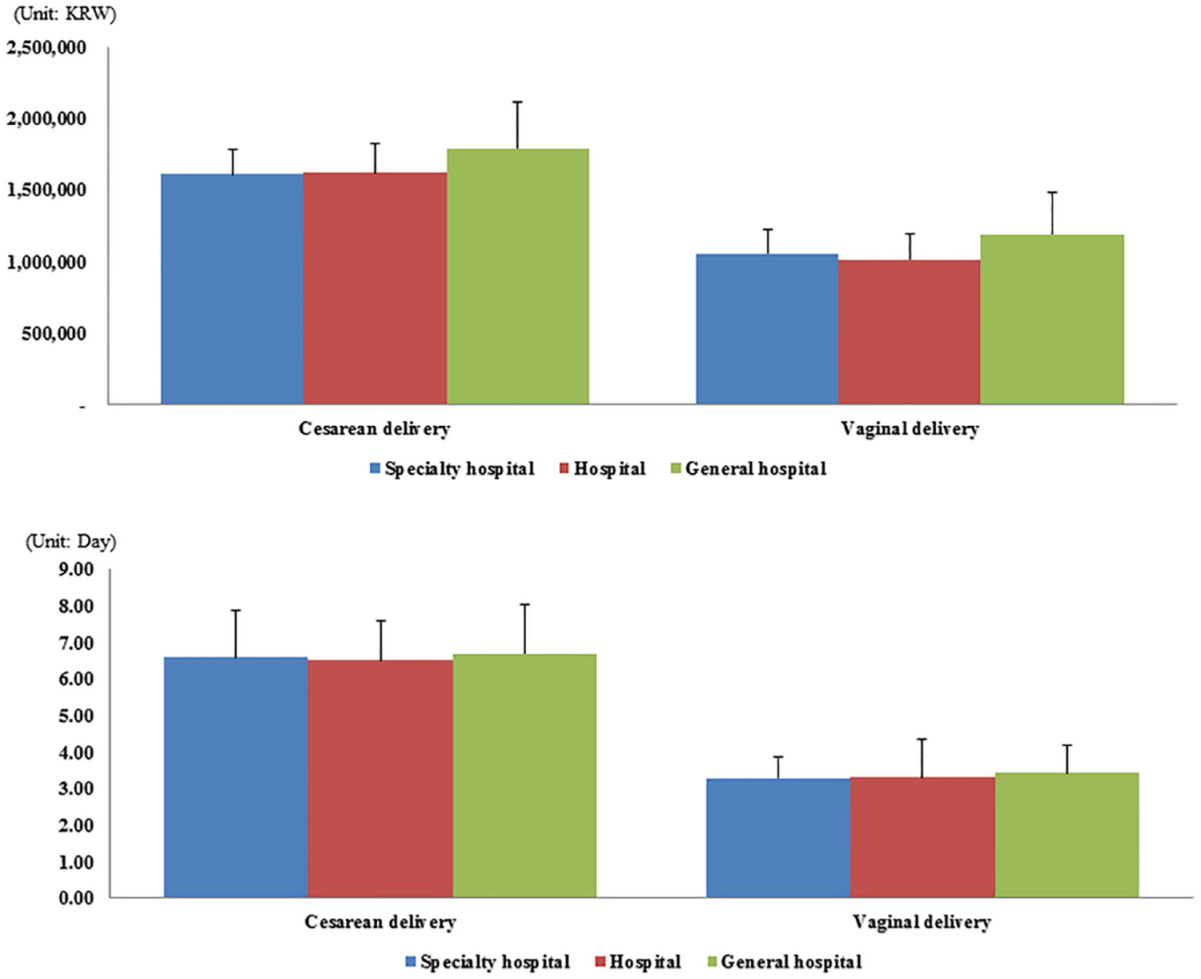
Differences in outcome variables between hospital type by Cesarean delivery and vaginal delivery. Data are shown as mean ± SD. All results are statistically significant by delivery type.

The average cost of CS deliveries was lowest in specialty hospitals (1,612,184; SD: ±177,132 KRW) and highest in general hospitals (1,791,112; SD: ±324,648 KRW). However, the average cost for vaginal deliveries was lowest in hospitals (1,014,056; SD: ±177,945 KRW) and highest in general hospitals (1,190,851, SD: ±289,832 KRW). Regarding average LOS, hospitals demonstrated the lowest average LOS for CS deliveries (6.51, SD: ±1.09 days), while specialty hospitals had the lowest LOS for vaginal deliveries (3.29, SD: ±0.57 days) (Fig 1).

The results of regression analysis with generalized estimating equation determined that the odds ratio of CS deliveries was lowest in specialty hospitals (OR: 0.95, 95% CI: 0.93–0.96), but the medical cost for CS deliveries was slightly higher in specialty hospitals (0.74%) compared with hospitals. The LOS for CS deliveries was higher (1%) in specialty hospitals compared with hospitals. With respect to vaginal deliveries, the medical cost increased (3%), but LOS decreased (0.57%) in specialty hospitals. In addition, the number of physicians and proportion of specialists were significantly associated with a low odds ratio for CS deliveries (10 doctors: OR- 0.99; proportion of specialists: OR- 0.99), low medical cost, and LOS in CS deliveries (Table 2).

**Table 2:** The regression analysis using with generalized equation estimating of outcome variable: Cesarean delivery, medical cost, length of stay.

								(Unit: OR, 95% CI/ Estimates, p-value)			
	Cesarean delivery			Cesarean section				Vaginal delivery			
	OR	95% CI		Medical cost		Length of stay		Medical cost		Length of stay	
**Type of hospital**											
Specialty hospital	0.95	0.93	0.96	0.0074	<.0001	0.0191	<.0001	0.0307	<.0001	-0.0057	<.0001
Hospital	1.00	-	-	Ref	-	Ref	-	Ref	-	Ref	-
General hospital	1.38	1.31	1.45	0.0619	<.0001	0.0992	<.0001	0.0411	<.0001	0.0150	<.0001
**Hospital location**											
Urban	0.66	0.60	0.73	0.0085	0.0011	-0.0998	<.0001	0.0315	<.0001	0.0254	<.0001
Rural	1.00	-	-	Ref	-	Ref	-	Ref	-	Ref	-
**Number of 100 Beds**	1.07	1.06	1.08	0.0022	<.0001	-0.0094	<.0001	0.0030	<.0001	0.0001	0.7782
**Number of 10 doctors**	0.99	0.99	0.99	-0.0001	0.4671	-0.0053	<.0001	0.0009	<.0001	0.0011	<.0001
Proportion of specialist*	0.99	0.99	0.99	-0.0001	<.0001	-0.0009	<.0001	0.0000	0.6827	-0.0003	<.0001
**Number of 10 nurses**	1.00	1.00	1.00	0.0000	0.532	-0.0016	<.0001	0.0006	<.0001	-0.0013	<.0001
**Proportion of patient**	1.01	1.00	1.01	0.0001	<.0001	-0.0015	<.0001	-0.0002	<.0001	-0.0003	<.0001
**Parity**											
Primipara	1.00	-	-	Ref	-	Ref	-	Ref	-	Ref	-
Multipara	1.25	1.23	1.26	0.0171	<.0001	-0.0252	0.0331	-0.1376	<.0001	-0.0638	<.0001
**Age**	1.05	1.05	1.05	-0.0012	0.4207	-0.0050	0.2163	0.0046	<.0001	-0.0001	0.3105
**LOS**				0.0254	<.0001			0.0873	<.0001		
**PCCL**											
0	1.00	-	-	Ref	-	Ref	-	Ref	-	Ref	-
1	1.84	1.81	1.87	0.0586	<.0001	0.0086	0.3166	0.0307	<.0001	0.0214	<.0001
2	4.10	3.98	4.22	0.2737	<.0001	0.0205	0.0165	0.0977	<.0001	0.0385	<.0001
3	5.89	5.20	6.67	0.4186	<.0001	0.0424	0.2378	0.2078	<.0001	0.0967	<.0001
**Year**											
2012.07~2013.06	1.00	-	-	Ref	-	Ref	-	Ref	-	Ref	-
2013.07~2014.07	0.97	0.96	0.98	0.0021	0.6245	-0.0299	0.0079	0.0447	<.0001	-0.0020	0.0015

* per 10% increased.

LOS: Length of Stay/ PCCL: patient clinical complexity level.

LOS: Estimates are the results of log transformation and interpretable as percentage changes.

Medical cost: Estimates are the results of generalized estimating equation with gamma distribution and interpretable as percentage changes.

-Note: All listed variables were entered simultaneously.

Table 3 shows the result of subgroup analysis by age groups indicated that the age under the 35 years old had a similar trend with main results. However, the age above 35 years old was different in the likelihood of CS. Both specialty hospitals and general hospitals have significantly low odds ratio of CS compared with hospital (specialty hospital: OR- 0.81/ general hospital: OR-0.78).

**Table 3:** Subgroup analysis of outcome variable by age group.

								(Unit: OR, 95% CI/Estimates, p-value)			
	Cesarean delivery			Cesarean section				Vaginal delivery			
	OR	95% CI		Medical cost		Length of stay		Medical cost		Length of stay	
**35**≤											
**Type of hospital**											
Special hospital	0.96	0.94	0.98	0.0069	<.0001	0.0206	<.0001	0.0317	<.0001	-0.0048	<.0001
Hospital	1.00	-	-	Ref	-	Ref	-	Ref	-	Ref	-
General hospital	1.50	1.42	1.60	0.0606	<.0001	0.0926	<.0001	0.0394	<.0001	0.0156	<.0001
**>35**											
**Type of hospital**											
Special hospital	0.81	0.78	0.86	0.0104	<.0001	0.0151	<.0001	0.0270	<.0001	-0.0132	<.0001
Hospital	1.00	-	-	Ref	-	Ref	-	Ref	-	Ref	-
General hospital	0.78	0.69	0.89	0.0696	<.0001	0.1185	<.0001	0.0372	<.0001	0.0119	0.1427

LOS: Estimates are the results of log transformation and interpretable as percentage changes

Medical cost: Estimates are the results of generalized estimating equation with gamma distribution and interpretable as percentage changes

- Note: Adjusted for type of hospital, hospital location, number of 100 beds, number of 10 doctors, proportion of specialist, number of 10 nurses, proportion of patient, parity, age, PCCL, and year.

## Discussion and conclusions

We wanted to assess the effects of specialty hospitals on CS delivery frequency and to provide policy-makers with evidence regarding performance of these hospitals with respect to associated medical costs and LOS in OBGYN care. Our results imply that specialty hospitals may not provide unnecessary procedures to patients, including CS, which is the most expensive method of delivery and may affect the physician’s choice of treatment. Previous studies have found that the cost of the procedure influences the decision to perform a CS [18,27,28]. Thus, CS deliveries provide an incentive to health providers for determining the method for delivery. However, vaginal delivery has been the recommended delivery method in Korea for reducing the high rate of CS deliveries, as well as lowering LOS and frequency of post-delivery complications. Specialty hospitals represent an effort to reduce unnecessary CS procedures because they have significantly higher numbers of specialized physicians and available health services. Thus, maintaining OBGYN specialization may lead to decreased CS delivery rates in specialty hospitals.

In our study, specialty hospitals were associated with a higher cost of delivery compared with other hospitals, likely due to greater incidences of intensive procedures [9,10] performed to improve the quality of care and reduce the frequency of post-delivery complications and readmission [29]. Thus, high levels of intensive care for patients increase medical cost in delivery but should be considered in context with the average patient LOS. Regarding LOS, specialty hospitals exhibited lower LOS for vaginal deliveries and increased LOS for CS deliveries compared with other hospitals. In general, CS required longer periods for recovery and was associated with more complications after delivery [30]. Because CS patients need more time and health services for recovery, their LOS in specialty hospitals is higher than in other hospitals. Increased LOS was directly associated with cost in CS cases because the hospitals are reimbursed by the DRG system. Although the cost for service is fixed under the DRG-based system, it does differ according to hospital LOS. Thus, increased LOS may be associated with medical cost in CS cases in specialty hospitals. With respect to vaginal delivery, recovery time was shorter than that of CS delivery. Thus, although specialty hospitals provide intensive care for briefer periods of time, this situation can still lead to high medical cost but shorter LOS. On the contrary, general hospitals had higher medical costs and longer LOS compared with other hospitals, regardless of delivery mode. These differences may result from the varying characteristics specific to each hospital type.

Further, the other covariates had some interesting findings or similar with previous findings. With respect to human resources variables, including specialists, higher proportion of specialist was associated with lower cost and LOS. It was because that such higher proportion could be positive role in improving efficacy of hospitals [31]. Consequently, if patients had same condition and visited hospital with similar structure, increasing the specialist would be getting better health outcomes. Thus, based on these results, healthcare professionals have to consider the optimal evaluation and reimburse for hospital with better staffing. In the results by doctors or nurses, there are common results in LOS because the higher staffing had better quality of care similar with previous studies [32]. These results imply that human factors have a significant impact on the quality of care and should maintain an adequate level of staffing to maintain quality. However, in the aspects of cost, there are slightly increased for cost that bigger sized hospital such as general hospital had higher additional rate rates under the NHI. Based on the clinical status of patients, patients with older age had higher cost in vaginal delivery. It was also caused by the additional rate that patients more than 35 years 30% additional paid for delivery by risk. By the severity, the patients with higher PCCL had generally higher cost and LOS, and it also similar with previous findings [33,34].

Our subgroup analysis revealed that the likelihood of CS in patients younger than 35 years old was lower in specialty hospitals and higher in general hospitals compared with other hospitals. We observed similar results in specialty hospitals for patients over 35 years old, although the relationship was more significant among the younger patients. General hospitals also had a lower likelihood of CS cases in patients over 35 years old, which could result from different characteristics of each type of hospital. For example, specialty hospitals have OBGYN specialists that can provide more appropriate health service to high-risk groups and perhaps decrease the likelihood of CS in these patients. In contrast, general hospitals may consider the economic benefit of CS deliveries; indeed, we observed a high odds ratio of CS deliveries in patients under 35 years old. These patients generally experience a lower risk of CS compared with older patients, yet the higher frequency of CS among younger women would lead to high-cost procedures whose reimbursement would provide significant profit. However, in the case of high-risk patients (greater than 35 years old), the patients’ medical conditions dictate the decision for CS deliveries and possibly lower the likelihood of CS procedures in general hospitals. Regarding medical cost and LOS, we observed similar trends based on hospital type, although LOS was shorter in specialty hospitals, regardless of patient age.

High competition among hospitals was introduced as a new strategy for improving their performance. As a result, specialty hospitals focus on specific clinical areas, provide high-quality care, and attempt to reduce medically unnecessary procedures. However, disputes regarding incentives for specialty hospitals to maintain their standard of care remain. In Korea, an incentive program exists for hospitals with better patient outcomes with respect to certain diseases (e.g., cancer, ischemic heart disease), outpatient services, and surgeries compared with other medical institutions. However, no incentive program for specialty hospitals is currently in place, which could negatively affect specialty hospitals, because, they are provided no additional benefits for efforts taken to maintain their specialty designation. Thus, policy-makers should consider providing additional financial or other related incentives to specialty hospitals that demonstrate high levels of performance. Such a reward could encourage these hospitals to strive for satisfactory patient outcomes and continue providing high-quality specialized care. However, the type of incentive should be considered carefully, and hospitals should be evaluated accurately and regularly by focusing on the relevant characteristics of specialty hospitals that influence their quality of care.

Our study has several strengths. First, we used the NHI claim data that included large sample sizes of both patients and hospitals; thus, our results should be of significance to policy-makers. Second, to the best of our knowledge, our study is the first to evaluate the relationship between specialty hospitals and rates of CS delivery, medical cost, and LOS in Korean medical institutions. Third, our results provide valuable evidence to policy-makers regarding the need for incentive programs aimed at helping specialty hospitals maintain their high standards of specialized patient care.

Despite these strengths, our study does have some limitations. Because we used NHI claim data, we were unable to measure patient socioeconomic status, such as income and education, which could affect the patients’ likelihood of a CS delivery. In addition, we did not consider maternal and neonatal clinical conditions, such as problems with placenta, abnormal bleeding, dysfunctional labor, and fetal distress during previous CS delivery. We also could not directly measure the effects of non-payment procedures on total medical costs among different hospital types. We did not examine other quality of care indicators, such as readmission rates and post-delivery complications, nor did we investigate other specialties beyond OBGYN. Thus, further studies are needed to evaluate the quality of care in specialty hospitals compared with other types of hospitals.

In conclusion, our results indicate that specialty hospitals are associated with a low likelihood of CS deliveries and short LOS after vaginal deliveries. Although specialty hospitals generate higher costs, they also provide better patient outcomes than other types of hospitals. Our findings highlight the need for an incentive program to maintain high levels of performance of specialty hospitals with respect to efficiency and patient care. However, regular evaluation and strict criteria for measuring performance are needed to account for other hospital characteristics; thus, additional studies that examine other factors influencing care at specialty hospitals may be helpful in developing such evaluation criteria.
